# Emergency preparedness knowledge and training needs among community volunteers in Shanghai, China, during the 2022 COVID-19 response: a cross-sectional questionnaire study

**DOI:** 10.3389/fpubh.2026.1973068

**Published:** 2026-10-05

**Authors:** Hao Zhang, Yuan Mei, Zhou Jiang, Jie Liu, Jin Li, Rui Zhang, Xu-jue Ma

**Affiliations:** 1 Department of Radiology, Dianjiang People's Hospital of Chongqing, Chongqing, China; 2 Department of Medical Administration, Dianjiang Health Commission of Chongqing, Chongqing, China; 3 Department of Radiology, First Affiliated Hospital of Chongqing Medical University, Chongqing, China; 4 Department of Orthopedics, The First Affiliated Hospital of Chongqing Medical and Pharmaceutical College, Chongqing, China; 5 Department of Emergency Medicine, The First Affiliated Hospital of Chongqing Medical and Pharmaceutical College, Chongqing, China; 6 Department of Medical Affairs, The First Affiliated Hospital of Chongqing Medical and Pharmaceutical College, Chongqing, China; 7 Department of Critical Care Medicine, The First Affiliated Hospital of Chongqing Medical and Pharmaceutical College, Chongqing, China

**Keywords:** community volunteers, COVID-19, emergency preparedness, health knowledge, needs assessment, personal protective equipment

## Abstract

**Background:**

Community volunteers support community-level emergency response, but in China they are typically mobilized on an *ad hoc* basis, without standardized entry requirements or systematic training. Their task-specific knowledge during actual public health emergencies has rarely been measured directly.

**Objective:**

This study assessed emergency preparedness knowledge and its demographic and training-related correlates among community volunteers during the 2022 COVID-19 response in Shanghai, China.

**Methods:**

A cross-sectional survey of community volunteers in Songjiang District, Shanghai, was conducted in May 2022 using convenience sampling. Knowledge was measured with an 18-item scale across five domains: COVID-19 transmission, hand hygiene, personal protective equipment (PPE) use, disinfection, and medical waste disposal; the maximum score was 18 points. Groups were compared using *t*-tests and one-way ANOVA with Bonferroni correction; multivariable linear regression identified independent correlates of the knowledge score.

**Results:**

Of 600 questionnaires distributed, 514 (85.67%) were analyzed after excluding 33 that were not returned and 53 that were incomplete. The mean knowledge score was 14.11 (SD 2.19) out of 18 points; item accuracy ranged from 30.54 to 99.81%, with eight of 18 items below the 75% mastery threshold. Accuracy was highest for medical waste disposal (94.70%) and lowest for PPE use (70.96%). Scores rose with educational attainment (*P* < 0.001) but not with age or training duration. Overall, 19.84% of volunteers received no training, and scores were higher with blended than with single-format training (mean 14.37 vs. 13.70–14.12; *P* = 0.049). Among trained volunteers, 49.39% considered training inadequate; the most requested topics were proper PPE use (77.63%) and infection control measures (60.51%). Multivariable regression (*n* = 485) confirmed educational attainment as the only independent correlate, with scores 1.1–1.9 points higher than in the group with a high school education or less (all *P* < 0.001); gender, age, and training duration were not independently associated with knowledge scores (all *P* ≥ 0.10).

**Conclusions:**

Emergency preparedness knowledge was uneven across domains: knowledge was adequate for medical waste disposal but below the mastery threshold for PPE use. About one-fifth of volunteers received no training. Educational attainment, not age or training duration, was the main independent correlate, and blended training was associated with higher scores. Education-stratified, hands-on training merits further study.

## Introduction

Public health emergencies threaten health, safety, and social stability worldwide. Communities are the first line of defense in such events. In this study, volunteers are defined as people who help during an event but are not part of the formal emergency management system. Those who live in and serve the same community are referred to as community volunteers and offer particular advantages: proximity to residents, familiarity with local conditions, and rapid mobilization (1). These advantages can supplement the capacity of government agencies and healthcare institutions during public health responses (2). Emergency preparedness is a pre-condition for effective response and refers to the knowledge, skills, and resources that individuals, institutions, and communities acquire in advance to prevent, respond to, and recover from public health emergencies (3). For community volunteers, preparedness is reflected mainly in whether they can carry out their assigned tasks safely and correctly. In China, community volunteers are usually recruited *ad hoc* by neighborhood committees during public health emergencies, with no standardized qualification requirements. Most receive only a brief pre-service orientation covering relevant theory and demonstrations of practical procedures, without return demonstration or skills testing, and then report directly to a neighborhood committee coordinator, who assigns tasks and supervises work on site. This *ad hoc*, non-standardized model of mobilization is common across the community emergency response system in China, and national regulations for managing volunteer preparedness are still lacking (4).

The 2022 COVID-19 outbreak in Shanghai created a substantial demand for community-level support. During the response, community volunteers assisted with nucleic acid sample collection, including guiding queues, maintaining order, and transporting specimens; delivered daily necessities; and helped maintain public order. These tasks placed them in frequent direct contact with residents and at relatively high risk of exposure. Because recruitment was rushed and training was limited, inadequate infection-prevention awareness and inconsistent protective practices were reported among volunteers (5, 6), reducing response effectiveness and increasing volunteers' own risk of infection (5). Experience in several countries indicates that systematic training programs help improve volunteers' knowledge and practical readiness (7). Existing assessments of emergency preparedness, however, have largely addressed the community or institutional level (8, 9), whereas research on community volunteers has focused mainly on service models and organizational management (4). The knowledge and training needs of individual volunteers for performing assigned tasks safely have rarely been measured empirically under actual emergency conditions.

To inform volunteer capacity building, we assessed emergency preparedness knowledge and training needs among community volunteers during the COVID-19 response in Songjiang District, Shanghai. Two questions were addressed. First, what was the overall level of emergency preparedness knowledge, and which items or domains fell below the mastery threshold? Second, which demographic characteristics and training experiences were associated with knowledge level? Unlike much earlier work, which relied largely on self-report or retrospective assessment, our data were collected during the acute phase of the outbreak in 2022, while volunteers were taking part in the response, allowing item-level measurement of knowledge during service and reducing recall bias. The gaps identified here are not specific to COVID-19 but may reflect broader weaknesses in the preparation of community volunteers in China, with relevance to other public health emergencies in the post-pandemic period. The findings may inform volunteer recruitment criteria, training programs, and community emergency response mechanisms, and may support the development of more resilient grassroots public health systems (10, 11).

## Methods

### Study design and ethics approval

This cross-sectional study was approved by the Institutional Review Board and was conducted in accordance with the Declaration of Helsinki. All participants were informed of the purpose of the study. Because the survey was anonymous, written informed consent was waived, and questionnaire completion was considered implied consent. In March 2022, two investigators joined a government-organized medical assistance team deployed to Songjiang District, Shanghai, to support the COVID-19 response. While on site, they identified problems in volunteer emergency preparedness and, with co-investigators, developed the study design and questionnaire. The on-site investigators used their position within the assistance team to liaise with local neighborhood committees and were responsible for pilot testing, questionnaire distribution, and answering participants' questions; co-investigators handled data checking, management, and statistical analysis. The two groups remained in contact through a working group on WeChat, a messaging and social media app widely used in China.

### Participants and setting

Community volunteers at the study site were recruited by convenience sampling from May 1 to May 31, 2022. The inclusion criteria were (1) being aged 18 years or older and willing to participate and (2) cumulative service in the COVID-19 response of 7 days or more. The exclusion criteria were (1) being a healthcare professional and (2) submitting an incomplete questionnaire. For the planned two-group comparison (gender), an *a priori* power analysis assuming a medium effect size (Cohen *d* = 0.5), 80% power, and α = 0.05 (two-sided) indicated a minimum of 63 participants per group [*n* = 2 × (1.960 + .84)^2^/0.5^2^ ≈ 62.7, rounded up to 63]. This value served as the overall benchmark for the study sample size. Allowing for the larger samples that independently powered multigroup comparisons (educational attainment, age group, training format) and their Bonferroni-corrected pairwise comparisons would require, together with anticipated non-response or invalid questionnaires, a total of 600 questionnaires were distributed.

### Instruments

Three instruments were used. The first was a demographic questionnaire, which collected gender, age, educational attainment, and duration of volunteer service. The second was an emergency preparedness knowledge scale developed from the national COVID-19 prevention and control protocol and relevant literature. Items were single-best-answer multiple-choice questions, each scored 1 point when answered correctly (maximum 18 points). Following common practice in emergency training assessment and consultation with five experts, a score of 75% correct or higher (i.e., 14 points or more) was defined as mastery. The 18 items covered five domains: routes of COVID-19 transmission, hand hygiene, personal protective equipment (PPE) use, environmental disinfection, and medical waste disposal. The third instrument was a training needs questionnaire, covering training experience, training format, and content needs. Training format was categorized, based on volunteers' self-report, as online only (all pre-service instruction delivered remotely, eg, via recorded lectures or livestreamed sessions), offline only (all instruction delivered in person), or blended (including both online and offline components). Training duration was the volunteer's own classification of total pre-service training days (no training, 1 day, or ≥2 days). Draft versions of the second and third instruments were reviewed by five experts in infection control, infectious diseases, and nursing, and item wording was revised accordingly to produce the final version. The item-level content validity index was not calculated. Before the main survey, the questionnaire was pilot-tested with 30 community volunteers to confirm item clarity and response logic. The full questionnaire, including all 18 knowledge items, response options, and the scoring key, is provided in Supplementary material S1.

### Data collection

After the pilot test with 30 community volunteers, the survey was administered online through Wenjuanxing (www.wjx.cn), a survey platform widely used in health research in China (12). With assistance from local neighborhood committee staff, the investigators posted the questionnaire and standardized instructions in the WeChat working groups used by community volunteers. The following quality control measures were applied: (1) questionnaires completed in less than 1 min were excluded as evidently inattentive responses, based on the pilot test completion-time distribution (*n* = 30; median 433 s, interquartile range 249–530 s, minimum 132 s); (2) responses to key items were checked for logical consistency; (3) response patterns and completion rates were monitored, and unusual questionnaires were flagged and rechecked; and (4) after export, data were checked independently by two investigators, who compared results.

### Statistical analysis

Data were analyzed with SPSS 25.0 (IBM Corp). Continuous variables are presented as mean (SD) and categorical variables as frequency and proportion. Age was grouped in 5-year intervals, with ages 46 years and older combined into 46–55 and ≥56 years because participant numbers declined steadily above 45 years. Knowledge scores were compared across groups (gender, educational attainment, age group, training duration, and training format) with independent-samples *t*-tests and one-way ANOVA; training format was analyzed only among volunteers who had received training (*n* = 412). Bonferroni correction was applied for multiple comparisons, and effect sizes were reported as Cohen *d*. Distribution shape was assessed with skewness, kurtosis, and the Shapiro-Wilk test, and variance homogeneity with the Levene test; parametric tests served as the primary analyses (13), with the Mann–Whitney *U* and Kruskal–Wallis *H*-tests as sensitivity analyses. A multivariable linear regression model was fitted with the total knowledge score as the dependent variable and gender, age (continuous), educational attainment, and training duration as independent variables; multicollinearity was assessed with the variance inflation factor (VIF). A total of 29 participants (5.6%) were excluded for missing age data; given this low proportion, complete-case analysis was used rather than imputation (14). Item-level accuracy was analyzed to identify specific knowledge gaps and training priorities. All tests were 2-sided, with α = 0.05.

## Results

### Participant characteristics

Of the 600 questionnaires distributed, 567 were returned (response rate 94.50%). After 53 incomplete questionnaires were excluded, 514 remained for analysis (90.65% of those returned; 85.67% of those distributed). Participant characteristics are shown in Table 1. Women and men were represented in approximately equal numbers; most volunteers were 40 years of age or younger, and 53.9% held a bachelor's degree or higher.

**Table 1:** Participant characteristics of community volunteers (*n* = 514).

Characteristic	Number	Percentage (%)
Gender		
Male	255	49.61
Female	259	50.39
Age, years^1^		
≤ 30	141	27.43
31–35	96	18.68
36–40	78	15.18
41–45	68	13.23
46–55	72	14.01
≥56	30	5.84
Missing	29	5.64
Educational attainment		
High school education or less	107	20.82
Associate's degree	130	25.29
Bachelor's degree	214	41.63
Graduate degree	63	12.26

^1^Age was recorded for 485 volunteers: mean 37.51 (SD 10.42) years, median 36 years, range 19–74 years. All percentages were calculated with 514 as the denominator.

### Emergency preparedness knowledge

The mean emergency preparedness knowledge score was 14.11 (SD 2.19) out of a maximum of 18 points. Item-level accuracy varied widely, from 30.54 to 99.81% (Figure 1). Seven items reached 90% accuracy or higher (three in the medical waste disposal domain). Eight items fell below the 75% mastery threshold; the weakest items concerned how often the outer surface of a mask was touched with bare hands (item 10, 30.54%), the procedure for nucleic acid testing (item 5, 50.00%), and the characteristics of the Omicron variant (item 2, 55.84%). At the domain level, accuracy was highest for medical waste disposal (94.70%), followed by environmental disinfection (82.39%). Three domains fell below the 75% threshold: routes of COVID-19 transmission (73.11%), hand hygiene (72.83%), and PPE use (70.96%), the lowest-scoring domain.

**Figure 1:**
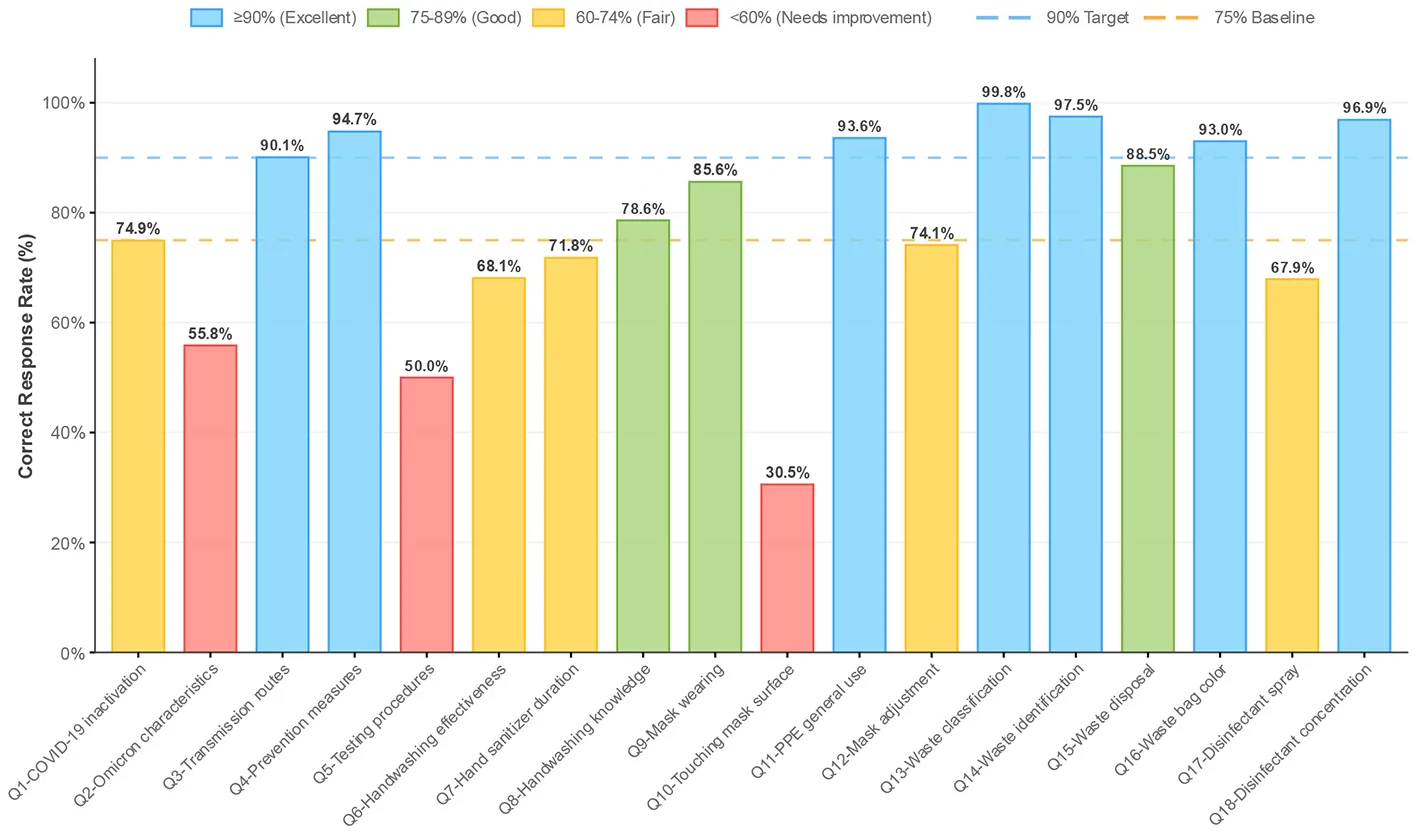
Emergency preparedness knowledge by individual item (*n* = 514). The abbreviated labels on the X-axis correspond to the 18 individual survey questions (Q1–Q18).

Items are ordered by knowledge domain: items 1–5, routes of COVID-19 transmission; items 6–8, hand hygiene; items 9–12, PPE use; items 13–16, medical waste disposal; and items 17–18, environmental disinfection.

### Differences in knowledge scores between groups

Women scored higher than men (mean 14.31, SD 2.26 vs. mean 13.92, SD 2.10; *t*_512_ = −1.99, *P* = 0.047), although the effect size was small (Cohen *d* = 0.18). Knowledge scores differed by educational attainment (*F*_3, 510 =_ 14.02, *P* < 0.001). Bonferroni *post hoc* comparisons showed that volunteers with an associate's, bachelor's, or graduate degree all scored higher than those with a high school education or less, with no significant differences among the three higher education groups (Figure 2). No significant differences were found across age groups (*F*_5, 479 =_ 1.66, *P* = 0.143) or training duration groups (*F*_2, 511 =_ 1.12, *P* = 0.326). Knowledge scores were negatively skewed (skewness−1.08, kurtosis 2.40; Shapiro-Wilk *W* = 0.92, *P* < 0.001), but variances were homogeneous within each grouping (Levene test: training duration *F*_2, 511 =_ 0.18, gender *F*_1, 512 =_ 0.43, age group *F*_5, 479 =_ 0.62, educational attainment *F*_3, 510 =_ 1.64; all *P* > 0.05). Sensitivity analyses with the Mann–Whitney *U*-test and the Kruskal–Wallis *H* test were consistent with the parametric results (gender *P* = 0.009, educational attainment *P* < 0.001, age group *P* = 0.146, training duration *P* = 0.268).

**Figure 2:**
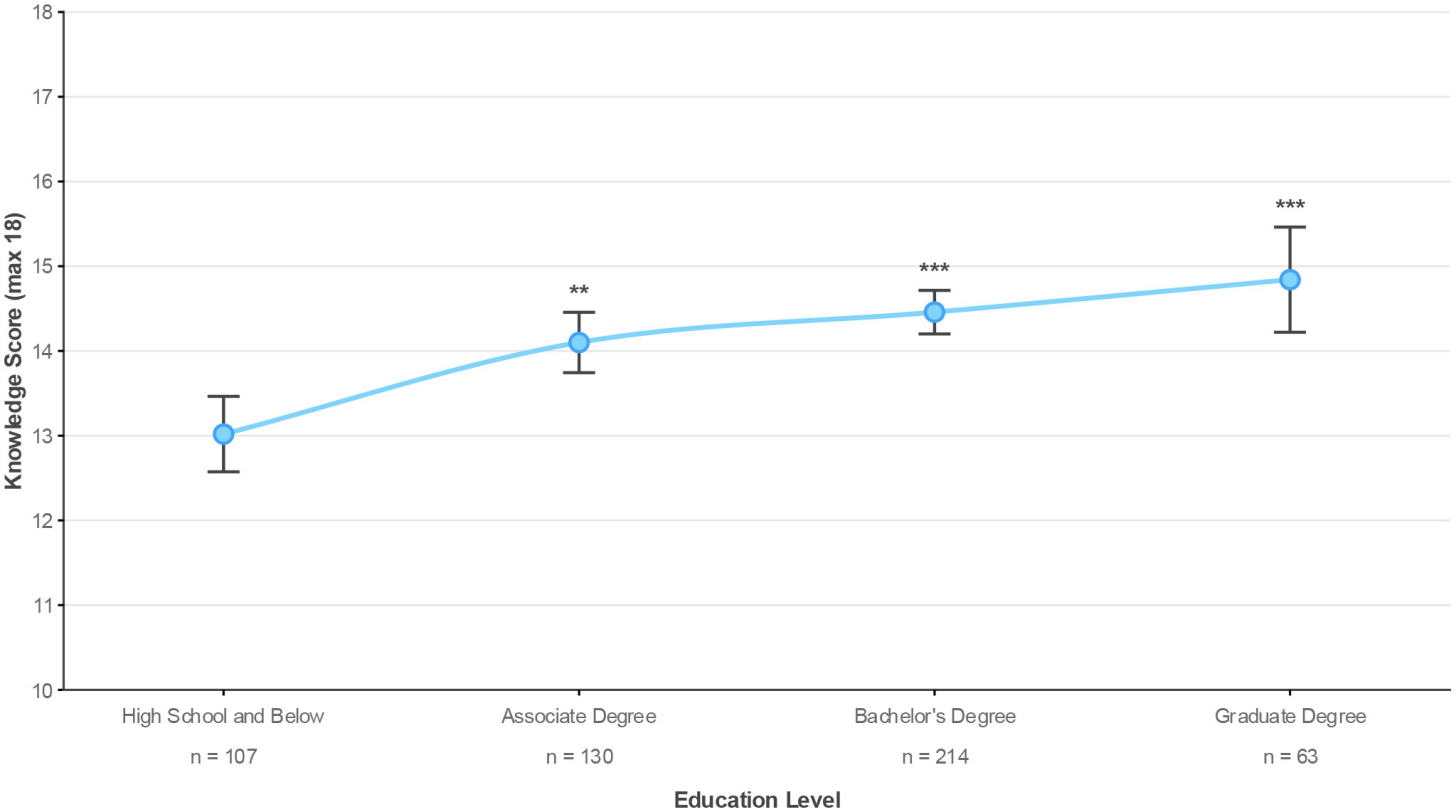
Emergency preparedness knowledge scores by educational attainment. Dots represent group means and error bars represent 95% CIs of the mean; the connecting line indicates the trend across successive education levels. **P < 0.01, ***P < 0.001 vs. high school education or less (one-way ANOVA with Bonferroni correction).

### Training status and training needs

Training status and training needs are shown in Table 2. A total of 102 volunteers (19.84%) began service without any training, and 204 (49.39%) of those who had been trained considered the content insufficient for their work. Knowledge scores differed by training format (*F*_2, 409 =_ 3.03, *P* = 0.049): scores were highest for blended online and offline training (mean 14.37, SD 2.07), followed by offline training only (mean 14.12, SD 2.09) and online training only (mean 13.70, SD 2.16). Educational attainment was not associated with the training modality received (Supplementary material 2 Table S1). Training needs centered on the proper use of PPE (77.63%) and COVID-19 infection control measures (60.51%).

**Table 2:** Training status and training needs of community volunteers (*n* = 514).

Item	Number	Percentage (%)
Training duration^1^		
No training	102	19.84
1 day	141	27.43
≥2 days	271	52.72
Training format (*n* = 412)		
Online	89	21.55
Offline	146	35.35
Online and offline	177	43.10
Training content was sufficient for assigned tasks (*n* = 412)		
Yes	208	50.49
No	204	49.51
Additional training content requested^2^ (*n* = 514)		
Proper use of personal protective equipment	399	77.63
COVID-19 infection control measures	311	60.51
Environmental surface disinfection	241	46.89
Medical waste disposal	151	29.38
Hand hygiene	79	15.37
COVID-19 epidemiology	47	9.14

^1^Training duration was counted in calendar days, with 8 h of instruction per training day; “≥2 days” denotes training that spanned 2 or more calendar days.

^2^Multiple responses were allowed; percentages were calculated with 514 as the denominator.

### Multivariable analysis of factors associated with knowledge score

Table 3 shows the multivariable linear regression model (*n* = 485 after exclusion of 29 participants with missing age data), with the total knowledge score as the dependent variable and gender, age, educational attainment, and training duration as independent variables. The overall model was significant (*F*_7, 477 =_ 6.53, *P* < 0.001) and explained 8.7% of the variance in knowledge score; VIFs ranged from 1.01 to 1.83, indicating no substantial multicollinearity. Educational attainment was the only factor independently associated with the knowledge score, with scores increasing across successive education levels: compared with volunteers with a high school education or less, scores were 1.085, 1.430, and 1.941 points higher among those with an associate's, bachelor's, and graduate degree, respectively (all *P* < 0.001). Volunteers trained for 2 days or more scored slightly higher than untrained volunteers, but the difference was not significant (*P* = 0.100). After adjustment, neither gender (*P* = 0.525) nor age (*P* = 0.372) was independently associated with the knowledge score.

**Table 3:** Multivariable linear regression analysis of factors associated with emergency preparedness knowledge scores among community volunteers (*n* = 485).

Variable	β	SE	95% CI	*P*-value
Intercept	12.469	0.466	11.553 to 13.385	< 0.001
Gender: male (ref: female)	−0.123	0.194	−0.504 to 0.258	0.525
Age (per year)	0.008	0.009	−0.010 to 0.026	0.372
Educational attainment: associate's degree (ref: high school education or less)	1.085	0.286	0.522 to 1.647	< 0.001
Educational attainment: bachelor's degree (ref: high school education or less)	1.430	0.263	0.912 to 1.948	< 0.001
Educational attainment: graduate degree (ref: high school education or less)	1.941	0.345	1.263 to 2.620	< 0.001
Training duration: 1 day (ref: no training)	0.103	0.287	−0.461 to 0.667	0.720
Training duration: ≥2 days (ref: no training)	0.427	0.259	−0.082 to 0.936	0.100

Model fit: R^2 =^ 0.087, adjusted R^2^^=^ 0.074; F_7, 477__=_ 6.53, *P* < 0.001. Reference categories: gender, female; educational attainment, high school education or less; training duration, no training.

## Discussion

Emergency preparedness knowledge among the 514 community volunteers was moderate overall, but this average masked variation across domains. Accuracy was highest for medical waste disposal (94.70%) and lowest for PPE use (70.96%), a domain closely tied to hands-on protective practice, and eight of the 18 items fell below the 75% mastery threshold. This pattern may reflect the attention given to waste management and environmental disinfection in recent community health education campaigns, and it points to gaps in practical skills requiring standardized training (5). A similar imbalance has been described in community health systems, where capacity building has focused on the institutional level with less attention to practical skills at the frontline (8, 9). Community volunteers have direct contact with residents and assist with tasks such as nucleic acid sample collection and supply distribution, so their exposure risk is relatively high. The gap in PPE knowledge and the finding that 19.84% of volunteers began service without any training therefore warrant attention. Existing evidence likewise indicates that adequate training and proper supervision are important for community volunteers to contribute to infectious disease surveillance and response (15).

Several factors were associated with volunteers' knowledge level. In univariate analysis, women scored higher than men (*P* = 0.047), although the effect size was small (Cohen *d* = 0.18); a survey of 990 registered disaster volunteers in Beijing reported a gender difference in the same direction (16). After adjustment for educational attainment and other covariates, gender was no longer significant (*P* = 0.525), suggesting that the apparent difference reflects differences in educational composition between male and female volunteers rather than an independent effect of gender. In multivariable regression, educational attainment was the only independent correlate of the knowledge score, with a graded association across education levels (Table 3). Training for community volunteers might therefore be tailored by educational attainment, with more demonstration and hands-on practice for volunteers who have had less formal education.

The regression model was significant (*P* < 0.001) but explained only 8.7% of the variance in knowledge score. *R*^2^ reflects a model's precision in predicting individual differences, a separate question from the reliability of its estimated associations (17). A low *R*^2^ therefore indicates limited prediction of individual scores rather than absence of association. In this study, training duration was not associated with the knowledge score, whereas the association with educational attainment was stable, suggesting that training content and delivery may deserve more attention than duration.

Training format was associated with knowledge scores. Volunteers who received blended online and offline training scored higher than those trained in a single format, consistent with reports supporting blended learning that combines theory with hands-on simulation (7). A supplementary analysis substituting training modality for training duration showed that combined training remained associated with the knowledge score after adjustment for educational attainment (Supplementary material 2 Table S2). Current volunteer training nevertheless had limited coverage and perceived adequacy. Comparable gaps exist in healthcare institutions: a survey of 803 health workers at 13 primary healthcare institutions in Wuhan found substantial differences in emergency response capacity across institutions and job types (18). The training needs reported here mirrored the knowledge gaps: PPE use (77.63%) and infection control measures (60.51%) were the most requested topics, both related to lower-scoring areas. International programs also use standardized, competency-based training; the Community Emergency Response Team (CERT) program of the US Federal Emergency Management Agency, for example, uses a nationally standardized tiered curriculum (19). A systematic review has similarly noted that hands-on skills training and simulation are often missing from conventional training programs (7).

These findings suggest several practical implications. Training should be stratified by educational attainment, with additional hands-on practice and supervised instruction for volunteers with less formal education. Training modules should focus on the weakest items identified in this study, including mask handling and the nucleic acid testing procedure, as well as the topics most frequently requested by volunteers. Blended training may be preferable because it allows online delivery of theoretical content and offline practice of operational skills. Training should also include feedback during service so that content can be adjusted as needs change, along with a system of routine continuing education. Future studies could use probability sampling, objective skills assessment, and process variables such as training content quality.

This study has several limitations. First, the study was conducted in a single district of Shanghai during a specific phase of the outbreak, which may limit generalizability. Second, convenience sampling may have introduced selection bias and overestimated overall preparedness. Third, the self-administered questionnaire is subject to social desirability bias, and knowledge scores do not fully reflect practical competence. Fourth, the cross-sectional design cannot establish a causal relationship between training and knowledge level. Fifth, the regression model did not include process variables such as training content quality or instructor qualifications, which may partly explain its limited explanatory power.

## Conclusion

This cross-sectional study showed that emergency preparedness knowledge among community volunteers in Songjiang District, Shanghai, was uneven across domains during the 2022 COVID-19 response. Accuracy was comparatively high for medical waste disposal but lower for practical infection-control skills, particularly PPE use. Educational attainment was the main independent correlate of knowledge level, whereas age, gender, and training duration were not independently associated with knowledge scores. Blended training was associated with higher scores. Nearly one-fifth of volunteers received no training before service, and nearly half of those trained still considered the content insufficient for their assigned tasks.

These findings support the development of standardized, education-stratified training programs that combine online instruction with offline, hands-on practice. Because of the cross-sectional design and the use of convenience sampling in a single district, both causal inference and generalizability are limited. Further studies using probability sampling and objective skills assessment are needed to confirm and extend these findings across broader regions and volunteer populations.

## Data Availability

The data analyzed in this study is subject to the following licenses/restrictions: the data that support the findings of this study are not publicly available due to participant privacy considerations but may be made available from the corresponding author upon reasonable request and with institutional review board approval. Requests to access these datasets should be directed to Hao Zhang, plutoye_7@hotmail.com.
