# Supplementary material for: Emergency preparedness knowledge and training needs among community volunteers in Shanghai, China, during the 2022 COVID-19 response: a cross-sectional questionnaire study
Source: Front Public Health. 2026 Oct 5;14:1973068. doi: 10.3389/fpubh.2026.1973068 (PMC13689685; doi:10.3389/fpubh.2026.1973068)
Supplement: Supplementary file 1 [file Table_1.docx]

**Survey on COVID-19 Protective Knowledge Among Community Volunteers**

*Songjiang District, Shanghai, China | May 2022*

*Supplementary Material S1 — Full Questionnaire*

**Part I. Participant Information**

Please provide your basic information in the table below.

| **Name** | **Age** | **Gender** | **Education Level** | **Community / Neighborhood Committee** |
| --- | --- | --- | --- | --- |
|  |  |  |  |  |

**Part II. Pre-Service Training Background (Self-Report)**

**1.** Did you receive COVID-19 infection prevention training before commencing volunteer service? **(Correct answer: —)**

A. No

B. Yes

**2.** If yes, what was the total duration of your training? **(Correct answer: —)**

A. Less than 1 day

B. 1 day or more

**3.** In what format was your training delivered? **(Correct answer: —)**

A. Online only

B. In-person (offline) only

C. Combined online and in-person

**4.** Do you feel the training adequately prepared you for your volunteer responsibilities? **(Correct answer: —)**

A. No

B. Yes

**5.** Which of the following topics would you most like additional training on? (Select all that apply)

A. COVID-19 epidemiological knowledge

B. COVID-19 infection prevention and control measures

C. Hand hygiene

D. Correct use of personal protective equipment (PPE)

E. Medical waste disposal

F. Environmental and surface disinfection

**Part III. COVID-19 Preparedness Knowledge Assessment**

Instructions: This section contains 18 single-choice questions. Select the single best answer for each question. Each correct answer scores 1 point (maximum total score: 18 points).

**Domain coverage:**

Questions 1–3: COVID-19 etiology and epidemiological characteristics

Questions 4–5: Standard precautions for COVID-19 prevention and control

Questions 6–8: Hand hygiene

Questions 9–12: Correct use of personal protective equipment (PPE)

Questions 13–16: Medical waste disposal

Questions 17–18: Environmental and surface disinfection

***Domain A — COVID-19 Etiology and Epidemiological Characteristics (Questions 1–3)***

**1.** Which of the following methods can effectively inactivate coronaviruses? (Single choice) **(Correct answer: E)**

A. Sensitive to ultraviolet (UV) light and heat

B. Heating to 56°C for 30 minutes

C. 75% alcohol

D. Chlorine-based disinfectant

**E. All of the above**

**2.** Which of the following is NOT a characteristic of the SARS-CoV-2 Omicron variant? (Single choice) **(Correct answer: D)**

A. Highly transmissible

B. Reduced pathogenicity compared with earlier variants

C. Shorter incubation period

**D. High rate of severe cases**

**3.** Which of the following is NOT a recognized transmission route of COVID-19? (Single choice) **(Correct answer: E)**

A. Respiratory droplet transmission

B. Aerosol transmission in relatively enclosed environments

C. Contact with virus-contaminated surfaces or objects

D. Close-contact transmission

**E. Bloodborne transmission**

***Domain B — Standard Precautions for COVID-19 Prevention and Control (Questions 4–5)***

**4.** Which of the following are recommended measures to prevent COVID-19 infection? (Single choice) **(Correct answer: E)**

A. Frequent handwashing and practicing cough etiquette

B. Wearing a mask correctly

C. Improving ventilation and performing environmental and surface disinfection

D. Avoiding crowding and maintaining social distancing

**E. All of the above**

**5.** During a community nucleic acid (PCR) testing session, which of the following behaviors is appropriate? (Single choice) **(Correct answer: D)**

A. Entry and exit pathways for the testing site do not need to be separated

B. Family members do not need to maintain a 1-meter distance from each other

C. Residents may remove their mask in advance to shorten the sample collection time

**D. Wearing a mask properly; brief greetings are acceptable while maintaining physical distance**

E. Wearing an isolation gown

F. All of the above

***Domain C — Hand Hygiene (Questions 6–8)***

**6.** How effective is handwashing in preventing the spread of pathogens, including SARS-CoV-2? (Single choice) **(Correct answer: D)**

A. No effect

B. Uncertain

C. Somewhat effective

**D. Very effective**

**7.** When performing hand hygiene with an alcohol-based hand rub, how long should the procedure generally take? (Single choice) **(Correct answer: C)**

A. Less than 5 seconds

B. 5–10 seconds

**C. More than 15 seconds**

**8.** Which of the following statements about handwashing is INCORRECT? (Single choice) **(Correct answer: C)**

A. Hands should be washed before touching the eyes, mouth, or nose

B. Hands should be washed after touching public facilities or surfaces

**C. Alcohol-based hand rub can completely replace handwashing with running water**

D. Handwashing follows a fixed technique and sequence that should not be replaced by personal habits

***Domain D — Correct Use of Personal Protective Equipment (Questions 9–12)***

**9.** When wearing a face mask, which of the following behaviors does NOT meet the required standard? (Single choice) **(Correct answer: B)**

A. A mask has a designated outward-facing side

**B. A mask only needs to cover the nose and mouth; a tight facial seal is unnecessary**

C. A mask must not be removed while speaking

D. A mask should not be reused

**10.** Have you ever touched the outer surface of your mask with your bare hands while on duty? (Single choice) **(Correct answer: A)**

**A. Never**

B. Occasionally

C. Frequently

** This item assesses behavioral compliance. "Never" (A) reflects the recommended safe practice and is the keyed correct answer.*

**11.** Which of the following statements about PPE use is correct? (Single choice) **(Correct answer: A)**

**A. Hand hygiene must still be performed even after putting on gloves**

B. PPE does not need to be changed during a work shift

C. The hood of the protective suit may be omitted if it obstructs vision

D. Conversing at close range or eating is acceptable when wearing full PPE

**12.** If your mask loosens or slips while you are on duty, what is the correct response? (Single choice) **(Correct answer: D)**

A. No replacement is needed; simply readjust the mask position

B. Replace the mask directly without additional precautions

C. Add a second mask layer over the existing one without prior hand hygiene

**D. Hold your breath, perform rapid hand hygiene, cover nose and mouth, then replace or add a protective mask layer**

***Domain E — Medical Waste Disposal (Questions 13–16)***

**13.** How should medical waste generated during COVID-19 volunteer service be classified? (Single choice) **(Correct answer: C)**

A. Ordinary household waste

B. Can be disposed of together with general household waste

**C. Potentially infectious; requires special handling**

D. Must be collected and stored separately from general waste

**14.** Which of the following items used during COVID-19 volunteer service are classified as medical waste? (Single choice) **(Correct answer: F)**

A. Protective suits

B. Gloves

C. Face shields

D. Face masks

E. Isolation gowns

**F. All of the above**

**15.** Which of the following statements about medical waste disposal is correct? (Single choice) **(Correct answer: B)**

A. PPE is not required when handling medical waste

**B. Medical waste bags must not be compressed, to prevent aerosol generation**

C. Once a bag is sealed, the outer surface does not require disinfection

D. Once sealed, medical waste bags may be placed in a general waste bin

**16.** What color bag is required for collecting medical waste in China? (Single choice) **(Correct answer: B)**

A. Black

**B. Yellow**

C. White

***Domain F — Environmental and Surface Disinfection (Questions 17–18)***

**17.** Is it acceptable to repeatedly spray disinfectant solution directly onto a protective suit while wearing it during volunteer work? (Single choice) **(Correct answer: B)**

A. Yes

**B. No**

**18.** Is it acceptable to prepare chlorine-based disinfectant at any concentration, with higher concentrations being preferable? (Single choice) **(Correct answer: B)**

A. Yes

**B. No**

**Answer Key**

**Correct answers:** Q1=E Q2=D Q3=E Q4=E Q5=D Q6=D Q7=C Q8=C Q9=B Q10=A Q11=A Q12=D Q13=C Q14=F Q15=B Q16=B Q17=B Q18=B

**Maximum score:** 18 points (1 point per correct answer). Higher scores indicate stronger COVID-19 protective knowledge.
