# Supplementary material for: Emergency preparedness knowledge and training needs among community volunteers in Shanghai, China, during the 2022 COVID-19 response: a cross-sectional questionnaire study
Source: Front Public Health. 2026 Oct 5;14:1973068. doi: 10.3389/fpubh.2026.1973068 (PMC13689685; doi:10.3389/fpubh.2026.1973068)
Supplement: Supplementary file 2 [file Table_2.docx]

**Supplementary Material 2**

**Table S1. Educational attainment by training modality among trained volunteers (*n* = 412)**

| **Educational attainment** | **Online**  **(n = 89)** | **Offline**  **(n = 146)** | **Combined online-and-offline**  **(n = 177)** |
| --- | --- | --- | --- |
| High school education or less | 28 (31.46) | 26 (17.81) | 38 (21.47) |
| Associate’s degree | 22 (24.72) | 42 (28.77) | 44 (24.86) |
| Bachelor’s degree | 28 (31.46) | 65 (44.52) | 77 (43.50) |
| Graduate degree | 11 (12.36) | 13 (8.90) | 18 (10.17) |

*Note.* Values are n (%) by column; *χ*^2^_6_ = 8.59, *P* = .198.

**Table S2. Sensitivity analysis substituting training modality for training duration in the multivariable model (*n* = 485)**

| **Variable** | ***β*** | **SE** | **95% CI** | ***P* value** |
| --- | --- | --- | --- | --- |
| Intercept | 12.511 | 0.465 | 11.596 to 13.425 | <.001 |
| Gender: male (ref: female) | −0.151 | 0.195 | −0.534 to 0.232 | .440 |
| Age (per year) | 0.008 | 0.009 | −0.010 to 0.027 | .364 |
| Educational attainment: associate’s degree (ref: high school education or less) | 1.061 | 0.286 | 0.499 to 1.623 | <.001 |
| Educational attainment: bachelor’s degree (ref: high school education or less) | 1.389 | 0.264 | 0.870 to 1.908 | <.001 |
| Educational attainment: graduate degree (ref: high school education or less) | 1.898 | 0.344 | 1.222 to 2.574 | <.001 |
| Training modality: online (ref: no training) | −0.123 | 0.322 | −0.755 to 0.509 | .702 |
| Training modality: offline (ref: no training) | 0.276 | 0.286 | −0.286 to 0.837 | .335 |
| Training modality: combined online-and-offline (ref: no training) | 0.552 | 0.274 | 0.013 to 1.091 | .045 |

*Note. R*^2^ = 0.094, adjusted *R*^2^ = 0.079; *F*_8,476_ = 6.20, *P* < .001; VIF 1.01–1.88.
